# An unsuspected role for organic cation transporter 3 in the actions of amphetamine

**DOI:** 10.1038/s41386-018-0053-5

**Published:** 2018-04-06

**Authors:** Felix P. Mayer, Diethart Schmid, W. Anthony Owens, Georgianna G. Gould, Mia Apuschkin, Oliver Kudlacek, Isabella Salzer, Stefan Boehm, Peter Chiba, Piper H. Williams, Hsiao-Huei Wu, Ulrik Gether, Wouter Koek, Lynette C. Daws, Harald H. Sitte

**Affiliations:** 1 Center for Physiology and Pharmacology, Medical University of Vienna, 1090 Vienna, Austria; 2 Department of Cellular and Integrative Physiology, University of Texas Health Science Center at San Antonio, San Antonio, TX 78229 USA; 3 Molecular Neuropharmacology and Genetics Laboratory, Department of Neuroscience and Pharmacology, Faculty of Health and Medical Sciences, University of Copenhagen, Panum Institute 18.6, 2200 Copenhagen N, Denmark; 4 Institute of Medical Chemistry, Medical University of Vienna, 1090 Vienna, Austria; 5 Department of Pediatrics, The Saban Research Institute, Children’s Hospital Los Angeles, Keck School of Medicine of USC, 4661 Sunset Blvd. Rm 307, Los Angeles, CA 90027 USA; 6 Department of Psychiatry, University of Texas Health Science Center at San Antonio, San Antonio, TX 78229 USA; 7 Department of Pharmacology, University of Texas Health Science Center at San Antonio, San Antonio, TX 78229 USA; 8 Center for Addiction Research and Science, Medical University Vienna, Waehringerstrasse 13 A, 1090 Vienna, Austria

## Abstract

Amphetamine abuse is a major public health concern for which there is currently no effective treatment. To develop effective treatments, the mechanisms by which amphetamine produces its abuse-related effects need to be fully understood. It is well known that amphetamine exerts its actions by targeting high-affinity transporters for monoamines, in particular the cocaine-sensitive dopamine transporter. Organic cation transporter 3 (OCT3) has recently been found to play an important role in regulating monoamine signaling. However, whether OCT3 contributes to the actions of amphetamine is unclear. We found that OCT3 is expressed in dopamine neurons. Then, applying a combination of in vivo, ex vivo, and in vitro approaches, we revealed that a substantial component of amphetamine’s actions is OCT3-dependent and cocaine insensitive. Our findings support OCT3 as a new player in the actions of amphetamine and encourage investigation of this transporter as a potential new target for the treatment of psychostimulant abuse.

## Introduction

Amphetamine-type drugs are among the most commonly abused in the world, afflicting an estimated 56 million people [1], creating a major public health burden, and underscoring a vital need to find effective treatments. Currently there are no medications to treat psychostimulant addiction, including addiction to amphetamine [2]. To develop effective treatments, the mechanisms by which these stimulants produce their abuse-related effects need to be fully understood. It is well known that amphetamine, as well as many other stimulants, interact with high-affinity transporters for monoamine neurotransmitters, the dopamine (DA), norepinephrine (NE), and serotonin (5-HT) transporters (DAT, NET, and SERT, respectively) [3]. These transporters are thought to be the main players regulating clearance of these monoamines from extracellular fluid. However, a rapidly growing literature supports a prominent role for organic cation transporter 3 (OCT3) in the regulation of monoaminergic neurotransmission [4–13].

OCT3 is a low-affinity (“uptake-2”) transporter with a high capacity to non-selectively transport monoamines [14]. It is also a bidirectional transporter, and there is evidence that OCT3 may play an important role in neurotransmitter efflux [5]. Moreover, gene variants of OCT3 have been linked to methamphetamine abuse [15]. OCT3 is distributed widely in brain, and is richly expressed in brain regions important in the rewarding effects of amphetamine, including striatum and nucleus accumbens [6, 12]. Taken together, these findings raise the possibility that OCT3 may be important for the actions of amphetamine-type psychostimulants that cause release of monoamines. However, whether OCT3 contributes to the actions of amphetamine is unclear.

It is well documented that amphetamine is a substrate for DAT, and therefore, a competitive inhibitor of DA uptake. Once inside the nerve terminal, amphetamine acts to increase cytosolic concentrations of DA via its activity at the vesicular monoamine transporter 2 (VMAT2), leading to reverse transport (or efflux) of DA into the extracellular space via DAT [16, 17]. A previous study reported that amphetamine is not a substrate for OCT3 and, therefore, concluded that amphetamine is unlikely to inhibit monoamine uptake via OCT3 [18]. However, it is conceivable that amphetamine, once gaining access to the intracellular compartment via its transport through DAT (or other mechanism(s)), may promote neurotransmitter efflux via OCT3. To our knowledge, no studies have investigated this possibility. Here we report the novel finding that a substantial component of amphetamine-evoked substrate release (i.e., DA and 1-methyl-4-phenylpyridinium (MPP^+^)) is OCT3-dependent and cocaine (a blocker of DAT, NET, and SERT) insensitive. Our findings encourage further investigation of OCT3 in the actions of amphetamine and as a potential target for the development of novel therapeutics to treat amphetamine addiction.

## Materials and methods

Wild-type mice (OCT3+/+), or OCT3 knockout (KO, OCT3−/−) mice, bred on a C57BL/6 background (originally developed by Zwart et al. 2001 [19]), were obtained from an in house colony at the University of Texas Health Science Center at San Antonio (UTHSCSA). Transgenic *Dat1*-eGFP mice were generated by the GENSAT project [20], and maintained at the University of Copenhagen. All procedures were conducted in accordance with the National Institute of Health Guide for the Care and Use of Laboratory Animals (Institute of Laboratory Animal Resources, Commission on Life Sciences, National Research Council 1996), and with the approval of the Institutional Animal Care and Use Committee, UTHSCSA, and the Danish Animal Experimentation Inspectorate (permission number: 2012-15-2934-00279). Details of age, gender, housing, and procedures using animals can be found in the Supplementary information.

OCT3 was identified in DAergic neurons using multiplex fluorescence in situ hybridization (FISH) in brain slices containing ventral tegmental area (VTA), or in DA neurons isolated from midbrain of *Dat1*-eGFP mice according to established methods [21, 22]. Amphetamine-stimulated locomotor activity was recorded using automated equipment. High-speed chronoamperometry was used to measure amphetamine-stimulated DA release in vivo, according to established methods [23]. Quantitative autoradiography of [^3^H]WIN35428 binding was used to assess DAT expression in OCT3+/+ and OCT3−/− mice. Radiotracer flux assays using [^3^H]MPP^+^ were carried out in rat cultured superior cervical ganglion cells (SCGs). The fluorescent substrate 4-(4-dimethylamino-styryl)-*N*-methylpyridinium (ASP^+^) or [^3^H]MPP^+^ were used to measure substrate uptake and efflux in human embryonic kidney (HEK293) cells expressing either human OCT3 tagged with yellow fluorescent protein (YFPhOCT3), hVMAT2, or hNET, according to established methods [24, 25]. Detailed descriptions of all methods can be found in the Supplementary information.

## Results

### OCT3 is expressed in DAT- and VMAT2-positive neurons

Since the primary action of amphetamine results in robust DA release, we first wanted to confirm that OCT3 is expressed in dopaminergic neurons.

**Fig. 1:**
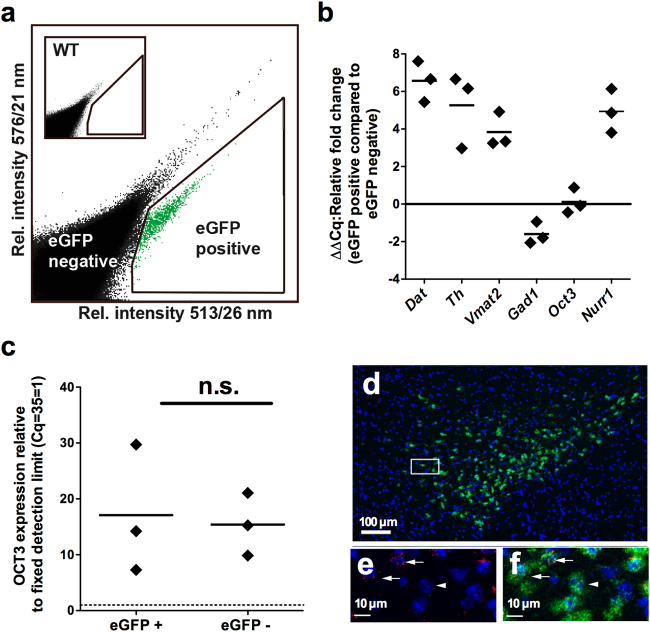
Identification of OCT3 in dopaminergic neurons and VMAT2-positive cells. **a** Fluorescence activated cell sorting (FACS) of a single cell solution of transgenic *Dat1*-eGFP midbrain cells, with the relative intensity of fluorescence at 513/26 nm against 576/21 nm after excitation at 488 nm. Inset shows similar FACS of a single-cell solution from a wild-type littermate. **b** Relative expression of DA and non-DA markers in the eGFP positive (eGFP+) sorted population compared with the eGFP negative (eGFP−) sorted population after normalization to housekeeping genes (ΔΔCq), dopamine transporter (*Dat*), tyrosine hydroxylase (*Th*), vesicular monoamine transporter 2 (*Vmat2*), glutamate decarboxylase 1 (*Gad1*), and nuclear receptor related 1 protein (*Nurr1*). Data are represented as mean obtained from three experiments. Data reveal enrichment of DA markers (*Dat, Th, Vmat2, Nurr1*) and a decrease in expression of the non-DA marker (*Gad1)* in the eGFP+ population. Organic cation transporter 3 (*Oct3*) was expressed in equivalent amounts in eGFP+ and eGFP− populations, as highlighted further in **c**, showing the expression of *Oct3* relative to a fixed detection limit with *C*_q_ = 35 set to 1. *Oct3* is detected in both populations (*n* = 3, dashed line = detection limit) with no difference in expression between the two populations (two-tailed Mann–Whitney test, *p* = 0.9, n.s. not significant). Importantly, the *Oct3* primer pair was designed to be intron-spanning, eliminating the possibility of amplification of genomic DNA, which was also evident in data that showed no signal in the no-RT samples. Melt curve analysis of the product showed amplification of a specific target (data not shown), and primer blast analysis of the *Oct3* primers showed only one product for the primer pair, that product being *Oct3*. **d**–**f** Expression of *Oct3 in Vmat2*-positive neurons in VTA. Multiplex fluorescent in situ hybridization (FISH) using RNAscope probes directed against *Vmat2* (green) and *Oct3* (red) performed on adult fresh frozen sections. In VTA, approximately 50% of *Vmat2*-positive cells were also positive for *Oct3*. Arrow points to a cell expressing both *Oct3* and *Vmat2*. Arrowhead points to a cell expressing *Vmat2* only. Blue: DAPI

To this end, we used a previously established method for isolation of midbrain DA neurons by fluorescence-activated cell sorting (FACS) on the basis of enhanced green fluorescent protein (eGFP) expression in dopaminergic neurons in transgenic *Dat1*-eGFP mice [21]. Analysis by qPCR clearly detected OCT3 mRNA in DA positive (eGFP+) neurons although mRNA was not enriched in these neurons compared with eGFP negative (eGFP−) cells (Fig. 1a–c). Additionally, as revealed by RNAscope, OCT3 was detected in VMAT2-positive cells in VTA (Fig. 1d–f). Interestingly, both approaches showed that OCT3 was also observed in cells that were not DA- (Fig. 1b, c) or VMAT2-positive (Fig. 1d–f), indicating the presence of OCT3 on neurons other than those positive for DA or VMAT, as well as non-neuronal expression. This finding is consistent with existing literature reporting both neuronal and glial expression of OCT3 [4, 9, 12, 26–28].

### Ability of decynium-22 to inhibit amphetamine-induced hyperactivity depends on availability of OCT3

**Fig. 2:**
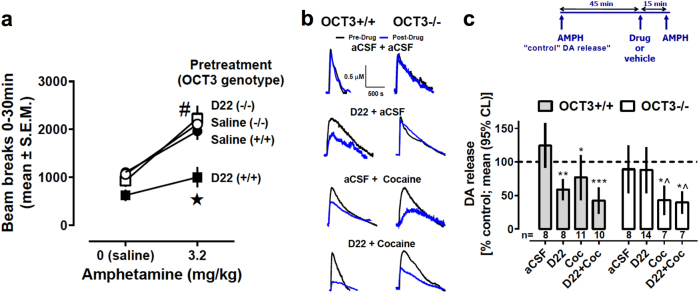
D22 attenuates amphetamine-evoked locomotor activity and DA release in OCT3+/+ mice, but not in OCT3−/− mice. **a** Amphetamine increased locomotion during the 30-min period immediately after an intraperitoneal (i.p.) injection of 3.2 mg/kg, and did so similarly in OCT3+/+ mice (filled circles) and in OCT3−/− mice (open circles). Pre-treatment with D22 (0.1 mg/kg) decreased this effect of amphetamine in OCT3+/+ mice (filled squares), but not in OCT3−/− mice (open squares). Results, shown as mean ± standard error of the mean (S.E.M, *n* = 7–8), were analyzed by two-factor ANOVA followed by multiple comparisons (Sidak’s test): *p < 0.05 compared with all other groups treated with 3.2 mg/kg amphetamine; ^#^*p* < 0.05 compared with saline-treated groups. Note that D22 did not significantly reduce locomotor activity in OCT3+/+ and OCT3−/− mice treated with saline instead of amphetamine (*p* = 0.87 and 0.99, respectively). **b**, **c** Intra-striatal pressure ejection of amphetamine (15 pmol) at 45 min intervals elicited reproducible oxidation currents that were identified as predominantly DA in nature, based on the ratio of reduction and oxidation currents produced (i.e., greater than 0.5; see Supplementary Table S3). Having established the reproducibility of amphetamine-evoked DA release in dorsal striatum of anesthetized mice, subsequent experiments followed the protocol of applying amphetamine (15 pmol), and 45 min later either aCSF vehicle, cocaine (15 pmol), or D22 (1 pmol), either alone or in combination, followed 15 min later by another application of amphetamine (15 pmol). **b** Representative oxidation signals produced by amphetamine-evoked DA release in striatum. Black traces are amphetamine-evoked DA release prior to aCSF or test drug; blue traces are amphetamine-evoked DA release 15 min following test drug, and are superimposed on pre-test drug traces for ease of comparison (see Supplementary Tables S1-3). **c** Time line showing that effects of test drugs and aCSF on amphetamine-evoked DA release were compared to the last release event that occurred prior to their administration. Summary data are shown as mean and 95 % confidence intervals (95% CL). Sample sizes for each group are shown below each column and ranged from 7 to 14. Data were analyzed by two-factor ANOVA followed by multiple comparisons (uncorrected Fisher’s LSD); *p < 0.05, **p < 0.001, ***p < 0.0001 vs. genotype-matched aCSF group; ^*p* < 0.05 vs. genotype-matched D22 group. In OCT3+/+ mice, there was no significant difference between D22+aCSF and D22+cocaine, but the difference between aCSF+cocaine and D22+cocaine approached significance (*p* = 0.058). Note that there was no significant difference between genotypes in response to cocaine (p = 0.09)

To determine if inhibition of OCT3 has consequences for the behaving animal, we investigated the effect of the non-selective OCT inhibitor, decynium-22 (D22), on amphetamine-induced locomotion in OCT3+/+ and OCT3–/– mice. Though D22 inhibits all OCT isoforms (OCT1, OCT2, OCT3), as well as the plasma membrane monoamine transporter (PMAT), its use for in vivo studies is preferential to the potent OCT3 blocker, corticosterone, which also has actions at glucocorticoid receptors [7]. Importantly, D22 does not appear to have any appreciable affinity for the high-affinity monoamine transporters (DAT, NET, and SERT) (unpublished data, manuscript in submission). Thus, the combined use of D22 with OCT3−/− mice affords a useful model to study OCT3-dependent actions of D22 and the consequences for behavioral response to amphetamine. Consistent with existing literature [12], there was no difference in basal locomotor activity between OCT3 genotypes under vehicle injection conditions. Maximal hyperactivity occurred after administration of 3.2 mg/kg amphetamine in OCT3+/+ and OCT3−/− mice (Supplementary Figure S1). Strikingly, however, pre-treatment with D22 decreased amphetamine-induced locomotion in OCT3+/+ mice but not in OCT3−/− mice (Fig. 2a). These effects of D22, which were observed during the first 30 min of the session, were also apparent during the entire session (Supplementary Figure S1) and suggest that OCT3 does indeed contribute to the actions of amphetamine.

### Ability of D22 to inhibit amphetamine-evoked DA efflux is dependent on OCT3 in vivo

Based on the ability of D22 to inhibit amphetamine-induced hyperlocomotion in OCT3+/+ mice, but not in OCT3−/− mice, we next investigated if pharmacological inhibition or genetic ablation of OCT3 would impinge upon the ability of amphetamine to evoke DA release in vivo. To this end, we used high-speed chronoamperometry to measure amphetamine-evoked DA release in striatum of OCT3+/+ and OCT3−/− mice (Fig. 2b, Supplementary Discussion and Tables S1–3). The striatum is not only rich in DAT, but also expresses OCT3 [6], and is important in the locomotor effects of amphetamine. There was no significant difference in amphetamine-evoked DA release between genotypes under control conditions, consistent with the lack of difference in amphetamine-induced hyperlocomotion. In addition, we found that expression of DAT (the primary target for amphetamine) in striatum, did not differ between genotypes (Supplementary Table S4).

Prior to testing drugs in vivo, we first confirmed in vitro that neither amphetamine, cocaine, D22, or their combination produced an electrochemical signal themselves, nor interfered with the recording properties of the electrode at the concentrations used here (see [13, 29]). As expected, amphetamine-evoked DA release in OCT3+/+ mice was inhibited (~23%) by pre-treatment with the DAT blocker, cocaine. However, D22 also inhibited (~41%) amphetamine-evoked DA release in OCT3+/+ mice, and when given together with cocaine produced greater inhibition (~58%) of amphetamine-evoked DA release than either drug given alone (Fig. 2b, c, Supplementary Tables S2, 3), suggesting that these drugs are acting at different sites. As for OCT3+/+ mice, cocaine inhibited (58%) amphetamine-evoked DA release in OCT3−/− mice. However, in contrast to OCT3+/+ mice, D22 had no effect on amphetamine-evoked DA release in OCT3−/− mice, nor did it enhance the effect of cocaine to inhibit (~60%) amphetamine-evoked DA release (Fig. 2b, c). Taken together, our in vivo data strongly support the idea that amphetamine-evoked DA release occurs via both DAT and OCT3.

**Fig. 3:**
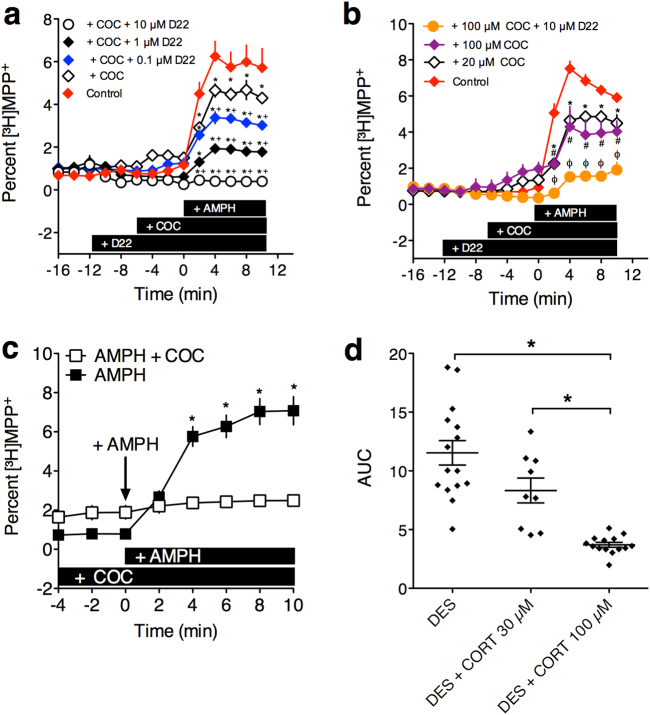
Amphetamine-induced efflux via OCT3 in an ex vivo system. **a** AMPH (10 µM) triggered efflux of pre-loaded [^3^H]MPP^+^ starting at *t* = 0 min from cultured rat superior cervical ganglia (SCG) cells in the absence or presence of cocaine (COC, 20 µM) or cocaine and D22 (COC 20 µM plus 0.1 µM D22, 1 µM D22, 10 µM D22, respectively). Addition of substances is indicated by black bars; Data are mean ± S.E.M., *n* = 9–12 independent observations per condition, *P < 0.05, (Bonferroni’s) compared to the control trace. **b** AMPH (10 µM) triggered efflux of [^3^H]MPP^+^ from SCGs (as in **a)** in the absence or presence of cocaine (20 or 100 µM, respectively) or cocaine (100 µM) and D22 (10 µM); * denotes statistical difference of COC 20 µM vs. the control trace; # denotes significance of COC 100 µM compared to the control trace and Φ indicates difference of COC 100 µM+10 µM D22 vs. all other groups (*, #, Φ, *P* < 0.05, Bonferroni’s). **c** AMPH-induced efflux of pre-loaded [^3^H]MPP^+^ starting at *t* = 0 min from HEK293 cells stably expressing human NET in the absence or presence of cocaine (COC; 20 µM). Black bars indicate addition of substances (*P < 0.05 (Bonferroni’s)). **d** AMPH (10 µM)-induced efflux of pre-loaded [^3^H]MPP^+^ from cultured SCGs (as in **a**) in the presence of desipramine (DES, 1 µM) and/or desipramine and corticosterone (CORT, 30 µM and 100 µM). For comparison, area under the curve (AUC) of the last five fractions in the presence of AMPH was calculated for each observation and analyzed by Kruskal–Wallis, followed by Dunn’s (*P < 0.05)

It is worth noting the relatively high degree of variance associated with most signal parameters in our chronoamperometric measurements (Fig. 2b). This is neither surprising nor unusual. A primary factor contributing to this variance is the excellent spatial resolution afforded by these small carbon fiber recording electrodes. Thus, the extracellular milieu surrounding the electrode (e.g., density of transporters) may differ significantly from experiment to experiment (i.e., from mouse to mouse), regardless of how consistent stereotaxic placement may be. Similarly, factors affecting diffusion through extracellular fluid (such as tortuosity and volume fraction) may also contribute to variance in release and clearance kinetics from one experiment to the next (compare representative pre-drug DA signals in Fig. 3a). Thus, our ability to detect marked effects of D22 and cocaine on amphetamine-evoked DA release in the face of this inherent “noise” underscores the magnitude and robustness of these drug effects on amphetamine-evoked DA release.

### Full inhibition of amphetamine-induced efflux of [^3^H]MPP^+^ in SCG cells ex vivo is only achieved when D22 is co-administered with a maximally effective concentration of cocaine

To further validate our in vivo findings, we turned to an ex vivo preparation of cultured rat SCG cells. SCGs are enriched with neuronal OCT3 and NET, but lack OCT1 or OCT2 [26]. By blocking NET with cocaine, this preparation provides a readily accessible *ex viv*o system to aid in deciphering the contribution of OCT3 to actions of amphetamine in neurons. SCGs were pre-loaded with [^3^H]MPP^+^, superfused with buffer, and exposed to amphetamine (10 µM), which resulted in a marked increase of substrate release (Fig. 3a). The addition of cocaine at a concentration sufficient to block amphetamine-induced efflux via NET (20 µM; Fig. 3b, c) failed to fully abolish amphetamine-evoked [^3^H]MPP^+^ release from SCGs, reducing the effect of amphetamine by only 30% (Fig. 3a). The cocaine-insensitive component of amphetamine-induced [^3^H]MPP^+^ efflux in SCGs was inhibited by D22, in a concentration-dependent manner (Fig. 3a). In the presence of cocaine (20 µM) and D22 (10 µM), amphetamine-evoked [^3^H]MPP^+^ efflux was completely blocked. Analysis by two-way ANOVA (treatment × time) revealed that drug treatments significantly affected the fractional release of [^3^H]MPP^+^ (*F*_4,38_ = 30.63, *P* < 0.0001). To ensure that 20 µM cocaine was fully blocking NET in SCGs, we increased the concentration of cocaine to 100 µM. As shown in Fig. 3b, the higher concentration of cocaine also inhibited amphetamine-evoked [^3^H]MPP^+^ efflux (two-way ANOVA, treatment × time, *F*_3,29_ = 17.08, *P* < 0.0001), but not to a greater extent than 20 µM, suggesting that 20 µM of cocaine is sufficient to fully block NET in SCGs. In line with this observation, 20 µM cocaine significantly inhibited amphetamine-induced [^3^H]MPP^+^ efflux in HEK293 cells stably expressing hNET (two-way ANOVA, treatment × time, *F*_1,25_ = 10.76, *P* < 0.01) (Fig. 3c).

It is not known if SCGs express PMAT. Therefore, to confirm that the effect of D22 to inhibit [^3^H]MPP^+^ release in SCGs is OCT3 dependent, we repeated this experiment using corticosterone in combination with the highly selective NET blocker desipramine. Corticosterone is a blocker of OCT3 [14] but not PMAT [30], and therefore is a useful tool to parse out the contribution of these transporters to substrate release. Like D22, corticosterone inhibited amphetamine-induced [^3^H]MPP^+^ release in the presence of a NET-inhibitor in SCGs, suggesting that the effect of D22 is unlikely to be PMAT dependent (Fig. 3d). Thus, consistent with our in vivo studies, ex vivo studies support a role for OCT3 in the actions of amphetamine.

### Amphetamine and cocaine are not inhibitors of OCT3-mediated transport of 4-(4-dimethylamino-styryl)-*N*-methylpyridinium (ASP^+^) in YFPhOCT3-expressing HEK293 cells

**Fig. 4:**
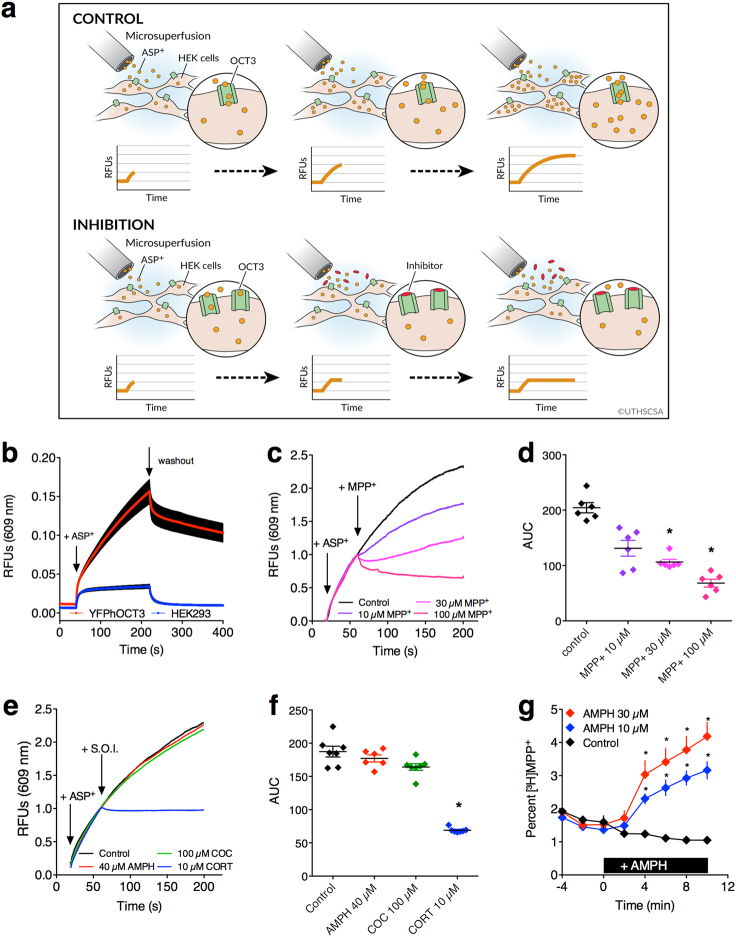
Amphetamine-induced efflux via OCT3. **a** Cartoon depicting experimental paradigm used for panels **b**–**f**. Under control conditions, microsuperfused ASP^+^ accumulates in YFPhOCT3 HEK293 cells leading to an increase in relative fluorescent units (RFUs) with time. Inhibitors of OCT3 block this accumulation. **b** Uptake of ASP^+^ in YFPhOCT3-expressing cells and parental HEK293 cells. Data are shown as mean ± S.E.M., *n* = 6–10 recordings per cell line. **c** Addition of MPP^+^ concentration-dependently inhibited uptake of ASP^+^ (arrows indicate the addition of ASP^+^ and MPP^+^). Shown are representative traces for each condition. For analysis, the area under the curve was calculated for each trace after the first 60 s. *P < 0.05, Kruskal–Wallis, followed by Dunn’s (**d**). **e** No detectable difference in ASP^+^ uptake upon addition of AMPH (40 µM) and cocaine (COC, 100 µM). Corticosterone (CORT, 10 µM) fully inhibited ASP^+^ uptake. Addition of ASP^+^ and the substance of interest (S.O.I.) is indicated by arrows. Shown are representative traces for each condition. For analysis, the area under the curve was calculated for each trace after the first 60 s. *P < 0.05, Kruskal–Wallis, followed by Dunn’s (**f**). **g** AMPH-induced efflux of pre-loaded [^3^H]MPP^+^ from YFPhOCT3 cells transiently transfected with hVMAT2 vs. vehicle-treated control. Addition of AMPH at *t* = 0 min is indicated by the black bar; Data are mean ± S.E.M., *n* = 9–12 independent observations, * indicates difference vs. control (*P < 0.05, Bonferroni’s)

Results so far implicate a role for OCT3 in the mechanism of action of amphetamine. However, because D22 is not selective for OCT3 over other OCT isoforms and PMAT, and because our in vivo studies do not afford deeper mechanistic insight into how the actions of amphetamine may be OCT3 dependent, we turned to a cell-based system to interrogate a role for OCT3 in the actions of amphetamine. To do so, we investigated OCT3-mediated transport of the fluorescent compound ASP^+^ into HEK293 cells stably expressing human OCT3 tagged with yellow fluorescent protein (YFPhOCT3) (Fig. 4a). ASP^+^ affords the temporal resolution to monitor instantaneous effects of test drugs on OCT3-mediated transport, providing a direct readout of substrate transport that is driven by the electrochemical gradient. ASP^+^ uptake was ~4.5-fold greater in YFPhOCT3 cells compared with parental HEK293 cells (Fig. 4b). Inversion of the electrochemical gradient for ASP^+^ (upon cessation of microsuperfusion of ASP^+^) instantly resulted in ASP^+^ release from YFPhOCT3 cells (Fig. 4b). Co-application of the non-fluorescent OCT3 substrate MPP^+^ competitively inhibited ASP^+^ uptake into YFPhOCT3 cells in a concentration-dependent manner (Fig. 4c).

It is well known that amphetamine, a substrate for DAT, and cocaine, a DAT blocker, potently inhibit uptake of substrates such as ASP^+^ and MPP^+^ via high-affinity monoamine transporters [3]. In contrast, neither amphetamine nor cocaine had any impact on OCT3-mediated uptake of ASP^+^ (Fig. 4d) and [^3^H]MPP^+^ (Supplementary Figure 2A), respectively, at concentrations below 40 µM. These data confirm that there is no direct action of amphetamine or cocaine on OCT3 at concentrations that have been reported to be behaviorally relevant [31–33]. In contrast, the OCT3 inhibitor, corticosterone, blocked uptake of ASP^+^ into YFPhOCT3 cells (Fig. 4d). Similar results were obtained with D22, which, like corticosterone, inhibited uptake of [^3^H]MPP^+^ (Supplementary Figure 2B). With both substances, half maximal inhibition was achieved with concentrations in the low micromolar range.

These results suggest that our in vivo and ex vivo findings are unlikely attributable to a direct action of amphetamine or cocaine at OCT3, as either a substrate or blocker. Importantly, if amphetamine is not a substrate for OCT3 (as our cell-based data, and others [18] show), amphetamine must gain entry to cells via an OCT3-independent mechanism.

### Co-transfection of YFPhOCT3 cells with hVMAT2 provides evidence that amphetamine gains access to the intracellular compartment via an OCT3-independent mechanism

Given our data showing that amphetamine does not gain entry to the intracellular compartment of YFPhOCT3 cells via OCT3, we wanted to determine that amphetamine can indeed gain entry to the cell. A prerequisite for amphetamine to induce reverse transport via the plasmalemmal transporters, DAT, NET, and SERT, is access to cytosolic monoamines. Converging lines of evidence suggest that amphetamine interacts with VMAT2, disrupting its function, and causing redistribution of monoamines from vesicles into the cytosol [17]. To determine if manipulations of VMAT2 function and concomitant elevations of cytosolic substrate result in outwardly directed transport via OCT3, we transfected YFPhOCT3 cells with hVMAT2. Cells were pre-loaded with [^3^H]MPP^+^, transferred into small chambers, and superfused. Addition of amphetamine (10 or 30 µM) significantly increased efflux of [^3^H]MPP^+^ compared to the untreated control condition (two-way ANOVA, treatment × time, *F*_2_,_27_ = 16.31, *P* < 0.0001). These data suggest that amphetamine is indeed able to gain access to the intracellular compartment of YFPhOCT3 cells, where it can act at hVMAT2 to increase cytosolic [^3^H]MPP^+^, resulting in gradient-driven reverse transport of [^3^H]MPP^+^ from the cells via OCT3 (Fig. 4e).

## Discussion

**Fig. 5:**
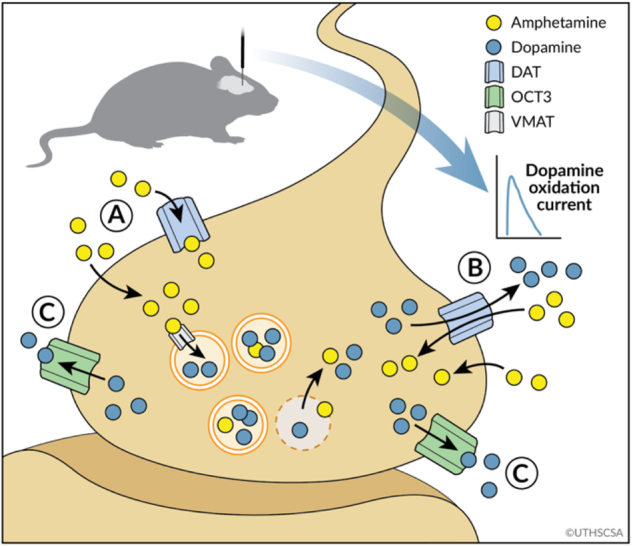
Model for OCT3-dependent amphetamine-evoked DA release. Cartoon depicting proposed mechanisms of action of amphetamine based on present results. Amphetamine enters neurons via DAT or diffusion across the plasma membrane “A”. Once inside, amphetamine causes release of DA via reverse transport through DAT “B” *and* OCT3 “C”

The major finding of the present study is that OCT3 is a previously unsuspected player in the complex mechanisms of action of amphetamine. This conclusion is based on our findings that (1) D22, the non-selective OCT/PMAT blocker, inhibited amphetamine-induced locomotor activity and amphetamine-induced DA release in vivo in wild-type mice, but not in OCT3−/− mice; (2) in ex vivo SCG preparations (rich in OCT3 and NET), a concentration of cocaine that fully blocks [^3^H]MPP^+^ efflux via NET, only fully blocked efflux in SCGs in the presence of D22. Furthermore, corticosterone augmented the inhibitory effects of the highly selective NET-inhibitor desipramine on amphetamine-induced release in SCGs; (3) as shown by our studies using YFPhOCT3 cells, while amphetamine is not a substrate for OCT3 (at least at behaviorally relevant concentrations), it can gain access to the intracellular compartment (putatively via diffusion across the plasma membrane) to interact with VMAT2 and subsequently promote efflux of substrate via OCT3. Figure 5 illustrates a working model of the OCT3- and DAT-dependent actions of amphetamine, based on these findings.

First it was important to verify if OCT3 is present on monoamine neurons in the central nervous system. Whether OCT3 resides on monoaminergic neurons remains under debate with evidence reported both for [9, 12, 26, 28, 34] and against [4, 27], at least with regard to DA neurons. Our cellular localization studies revealed OCT3 mRNA in isolated DAergic neurons (eGFP+). In addition, anatomically resolved RNAscope studies identified OCT3 in VMAT2-positive neurons in VTA. These findings are in line with others reporting neuronal localization of OCT3 [9, 12, 26, 28]. We also found OCT3 in DA-negative (eGFP−) and VMAT2-negative cells. This finding is in agreement with reports indicating non-neuronal expression of OCT3 [4, 27]. Thus, OCT3 might play a dual role in neuronal recycling and non-neuronal breakdown of DA in glial cells. This notion is further supported by recent electron microscopy studies revealing the presence of OCT3 on both neurons and glia in basolateral amygdala [34]. Taken together with results reported herein, these findings also raise the possibility that amphetamine might have previously unsuspected actions in non-neuronal cells.

Having confirmed the presence of OCT3 in DA neurons, we sought to examine the functional relevance of OCT3 for actions of amphetamine. Our first approach was to investigate the consequences of pharmacological inhibition of OCT3 on amphetamine-induced locomotion and DA release in vivo. To this end, we used D22, currently the best available blocker of OCT3, which, unlike corticosterone, lacks activity at glucocorticoid receptors. However, because D22 also inhibits OCT1, OCT2, and PMAT, we carried out these studies in both OCT3+/+ and OCT3−/− mice. D22 attenuated amphetamine-induced locomotion and DA release in OCT3+/+ mice, an effect that was lost in OCT3−/− mice. Moreover, D22 appeared to enhance (*P* = 0.058) the ability of cocaine to suppress amphetamine evoked DA release in OCT3+/+ mice, but not in OCT3−/− mice. Notably, compared to vehicle treated control OCT3+/+ mice, the combination of D22 and cocaine produced the most robust inhibition of amphetamine-evoked DA release (*P* < 0.0001) compared to either D22 (*P* < 0.001) or cocaine (*P*< 0.05).

Our behavioral data are consistent with those of Vialou and co-workers [12] who also found no difference in basal locomotor activity between OCT3 genotypes or in hyperlocomotion following 3.0 mg/kg amphetamine. However, Vialou et al. [12] reported that a higher dose (10 mg/kg) that did not increase locomotion in OCT+/+ mice (due to the emergence of stereotyped behaviors) did so in OCT3−/− mice. This suggests that the descending limb of amphetamine’s dose–response curve to increase locomotion was shifted to the right in OCT3−/− mice compared with OCT3+/+ mice (i.e., OCT3−/− mice are less sensitive to the effects of amphetamine), consistent with a role for OCT3 in the locomotor effects of amphetamine. Our results do not provide evidence of such a shift: 10 mg/kg did not increase locomotion, neither in OCT3+/+ nor in OCT3−/− mice, during the time of the session when amphetamine-induced locomotion was maximal (Supplementary Figure S1 B–D). Differing conditions for the locomotor assays used between our study and that of Vialou and coworkers (2008) likely account for the differing results. Regardless, the absence of effect of D22 on amphetamine-induced locomotion in OCT3−/− mice provides strong support for a role of OCT3 in actions of amphetamine.

Our results are consistent with reports that OCT3−/− mice do not differ in basal locomotor activity or show any overt behavioral phenotype [12, 35]. Keeping in mind that OCT3 is a *low-affinity, high-capacity* transporter for DA (and other monoamines), this is not a surprising result. For example, if DAT, the high-affinity, low-capacity transporter for DA, remains functionally unimpaired, as our results suggest, it is reasonable to expect no or very subtle phenotypes in OCT3−/− mice (as previously reported [12, 35]). The possibility remains that compensation in other monoamine transporters, including D22-sensitive transporters (e.g., OCT1, 2 and PMAT), might also contribute to the lack of an overt behavioral phenotype; however, we think this is unlikely based on constitutive PMAT−/− mice showing no changes in the expression of DAT, SERT, NET, or of OCT1, OCT2, or OCT3 [36]. That PMAT expression, like OCT3, is especially rich in brain adds further support to this contention. Moreover, our finding that the effects of D22 to inhibit amphetamine-induced locomotion and DA release were lost in OCT3−/− mice strongly suggests that these actions of D22 are not mediated via OCT1, OCT2, or PMAT. That OCT3+/+ and OCT3−/− mice show similar responses to amphetamine-induced locomotion and DA release, with a role for OCT3 *only being revealed by treatment with D22*, is therefore also not surprising (i.e., because OCT3−/− mice have a full complement of the high-affinity DAT). The exciting implication of our findings is that OCT3-dependent actions of amphetamine may provide a mechanistic basis for why DAT, SERT, and NET based therapeutics for amphetamine addiction have not been successful [2]. That is, OCT3-dependent actions of amphetamine may only become apparent when “uptake-1” transporters (DAT, NET, and/or SERT) are inhibited, and/or, when OCT3 is pharmacologically blocked. It is also worth noting that the pharmacokinetics and disposition of amphetamine do not differ between genotypes [18], which could also contribute to the lack of difference between genotypes in their locomotor response to systemically delivered amphetamine.

The unsurprising lack of any overt phenotype resulting from constitutively knocking out a low-affinity, high-capacity transporter, is further underscored by the dramatic phenotype resulting from knockout of the high-affinity, low-capacity, DAT. DAT−/− mice are hyperactive and show dampened or no response to DAT targeting drugs. However, consistent with our findings, amphetamine is not without effect in DAT knockout mice. Though the first characterization of these mice suggested that DAT knockout mice were insensitive to amphetamine [37], subsequent studies have shown that amphetamine does increase extracellular DA in DAT knockout mice [38], even though DA content in neurons of these mice is reduced 20-fold compared to wild-type mice (i.e., DAT knockout mice have less than 10% of total tissue DA [39]). In addition, the ability of amphetamine to influence electrically evoked DA release in ex vivo nucleus accumbens slice preparations has been reported to be both DAT dependent and DAT independent, supporting the notion that other mechanisms are at play [32]. These DAT-independent mechanisms are likely many (and may include SERT, NET, and/or receptors that regulate DA release), however, our data suggest that OCT3 may be one such mechanism. Regardless of potential compensation in other systems in OCT3−/− mice, our finding that the ability of D22 to inhibit amphetamine-induced locomotion and DA release is lost in OCT3−/− mice provides strong support for a role of OCT3 in the action of amphetamine.

Given that D22 was without effect in OCT3−/− mice, the most straightforward interpretation of the ability of D22 to suppress amphetamine-evoked DA release, and to enhance the ability of cocaine to do so in OCT3+/+ mice, is that D22 simply prevents efflux of DA via OCT3 by blocking this transporter. This interpretation is supported by studies suggesting that D22 is an antagonist at OCT3 [14, 40]. However, there is some evidence that D22 may accumulate into astrocytes via an OCT3 dependent mechanism [41], although this finding awaits further experimental verification. Hence, we cannot rule out the possibility that D22 may gain entry into DA neurons via OCT3 where it may interact with intracellular components, which in turn could interact with mechanisms that support amphetamine-induced DA efflux via OCT3-dependent, or non-OCT3-dependent mechanisms. Our results in OCT3−/− mice do not allow us to rule out this possibility. However, our finding that D22 augments the ability of cocaine to inhibit DA efflux in OCT3+/+ mice argues that, even if D22 is a substrate for OCT3, it is not driving some facet of signaling needed to support DAT-mediated DA release.

In terms of our in vivo chronoamperometry studies, it is also important to note that the concentration of amphetamine (400 μM) loaded into the micropipette, was selected so as to yield concentrations at the recording electrode of approximately 2–40 μM, taking into account well-documented dilution effects due to diffusion through extracellular space [23]. Previous studies have shown that this concentration range is consistent with those reported in brain after systemic administration of a behaviorally effective dose of amphetamine and its derivatives [31, 42]. Thus, the concentrations of amphetamine used our in vivo efflux experiments are behaviorally relevant and not supra-physiological.

Our in vivo findings are further supported by functional ex vivo studies performed in SCG cultures, which are rich in NET and OCT3 [26]. As expected, there was marked efflux of pre-loaded [^3^H]MPP^+^ following addition of amphetamine. However, addition of cocaine at a concentration that fully inhibits NET in cell-based systems failed to fully ablate the effect of amphetamine to induce [^3^H]MPP^+^ release from SCG cells. These data are consistent with our in vivo data and suggest that amphetamine-evoked [^3^H]MPP^+^ efflux occurs through mechanisms other than NET in SGCs. We found that full inhibition of amphetamine-evoked [^3^H]MPP^+^ release could only be accomplished if both OCT3 and NET were fully inhibited (i.e., by D22 and cocaine, respectively). Thus, cocaine may fully attenuate amphetamine-induced efflux of [^3^H]MPP^+^ via its action at NET (as well as DAT and SERT) (see Fig. 3 and [43, 44]) in cell-based expression systems, but in an ex vivo SCG cell preparation this inhibition is not complete. SCGs are rich in NET and OCT3, and devoid of OCT1 and OCT2 [26], supporting a role for OCT3 in the action of D22 to inhibit substrate efflux. It is unknown if PMAT is expressed in SCGs; however, our finding that corticosterone (which blocks OCT3 but not PMAT) recapitulated the effect of D22 to inhibit amphetamine-evoked [^3^H]MPP^+^ release in SCGs provides strong evidence that this effect of D22 is OCT3 dependent.

After replicating the well-established finding that cocaine does not affect OCT3-mediated transport [14], we found that D22 (OCT/PMAT inhibitor) enhances the ability of cocaine (DAT/NET/SERT blocker) to inhibit amphetamine-evoked substrate (DA, [^3^H]MPP^+^) release, both in vivo and ex vivo. Remarkably, however, we found that amphetamine does so in a manner that does not involve its transport into the cell via OCT3. Behaviorally relevant concentrations of amphetamine did not inhibit uptake of ASP^+^ into YFPhOCT3 expressing HEK293 cells, suggesting that amphetamine is not a substrate for OCT3, or at best, only a poor substrate at very high (supra-behaviorally relevant) concentrations. These findings, together with evidence that amphetamine can gain entry to cells via active transport [45] or diffusion across the plasma membrane [46–48], highlights access to cytosolic monoamines as a key element for OCT3 to contribute to the actions of amphetamine. Given that an important part of the mechanism of action of amphetamine is its activity at VMAT2, which results in release of monoamines from vesicular stores into the cytosol [17, 49], this notion is further supported by our data showing that amphetamine was able to evoke robust efflux of [^3^H]MPP^+^ in YFPhOCT3 cells co-transfected with hVMAT2. Interestingly, Freyberg et al. [49] reported that the effects of methamphetamine on locomotion were drastically reduced by acute treatment with a VMAT2 inhibitor, whereas the effects of cocaine were not affected. This finding supports the idea that the effects of amphetamine-related drugs at VMAT2 are essential to their action [49]. Perturbed VMAT2 function and concomitant elevations in cytosolic monoamines results in a gradient that favors outward transport (efflux). Since the electrochemical gradient of substrate provides the driving force for OCT3-mediated transport, our data are in accord with the notion that OCT3-dependent amphetamine-induced monoamine release is due to access of OCT3 to high concentrations of cytosolic monoamines. It is important to mention that earlier studies demonstrated that amphetamine induces release of monoamines that are derived from a “newly synthesized” pool in vivo [50]. Consistent with this observation, amphetamine was found to induce efflux of DA when vesicular stores were depleted with reserpine [51, 52]. Contrasting findings have been reported by Florin and colleagues, showing that in vivo effects of amphetamine are sensitive to reserpine [53]. Additionally, Freyberg et al. demonstrated that locomotion stimulated with methamphetamine and amphetamine was sensitive towards a VMAT inhibitor in a dose-dependent manner [49]. This assembly of pharmacological evidence indicates that alternative intracellular effects of amphetamine may contribute to OCT3-mediated reverse transport in vivo, ranging from inhibition of monoamine oxidases to activation of kinases and alterations of the transciptome [17, 54].

Taken together, our data support the working model depicted in Fig. 5. Amphetamine enters neurons via DAT (NET or SERT) or diffusion across the plasma membrane or perhaps even gaining entry into DA neurons via an as yet unknown mechanism (e.g., [55]). That amphetamine may gain entry by diffusion or via yet unknown mechanisms is supported by our finding that (1) pre-treatment of SCG neurons with cocaine at a concentration that fully blocks high-affinity monoamine transporters in cell systems failed to fully block amphetamine-evoked release of [^3^H]MPP^+^ in cultured neurons, and (2) amphetamine is, at best, a poor substrate for OCT3. Once inside the neuron, amphetamine reverses VMAT activity, causing release of DA into the cytosol and subsequent release of DA via reverse transport through DAT and OCT3. It must be kept in mind, however, that in addition to the intraneuronal effects of amphetamine discussed above, non-neuronal OCT3 might also contribute to the overall actions of amphetamine, through currently unknown mechanisms (if at all). It is possible that OCT3 and DAT may need to physically interact in order for amphetamine to promote efflux via OCT3. These will be important areas for future research.

In sum, our results reveal OCT3 as an important player in the actions of amphetamine. Current treatments for addiction to amphetamine and amphetamine-type psychostimulants targeting only the high-affinity transporters DAT, NET, and SERT are far from being satisfactory. Our results introduce OCT3 as a potential new target for the development of novel therapeutics to treat addiction to amphetamine and its congeners. Together, these findings nourish the field of psychostimulant research by pointing to OCT3 as an important player in harmonizing monoaminergic signaling.

## Electronic supplementary material

SUPPLEMENTAL MATERIAL
