# Supplementary material for: An unsuspected role for organic cation transporter 3 in the actions of amphetamine
Source: Neuropsychopharmacology. 2018 Apr 6;43(12):2408–17. doi: 10.1038/s41386-018-0053-5 (PMC6180071; doi:10.1038/s41386-018-0053-5)
Supplement: Supplementary file 1 — SUPPLEMENTAL MATERIAL [file 41386_2018_53_MOESM1_ESM.docx]

**Supplementary Information**

**An unsuspected role for organic cation transporter 3 in the actions of amphetamine**

**Authors:** Felix P. Mayer, PhD^a^, Diethart Schmid, MD^a^, W. Anthony Owens^b^, Georgianna G. Gould, PhD^b^, Mia Apuschkin, MSc^c^, Oliver Kudlacek, PhD^a^, Isabella Salzer, PhD, MD^a^, Stefan Boehm, MD^a^, Peter Chiba, MD^d^, Piper H. Williams^e^, Hsiao-Huei Wu, PhD^e^, Ulrik Gether, DMSc^c^, Wouter Koek, PhD^f,g^, Lynette C. Daws, PhD*^b,g^, Harald H. Sitte, MD*^#a,h^

**Affiliations:**

^a^Center for Physiology and Pharmacology, Medical University of Vienna, 1090 Vienna, Austria.

^b^Departments of Cellular and Integrative Physiology, ^f^Psychiatry and ^g^Pharmacology, University of Texas Health Science Center at San Antonio, San Antonio, TX 78229, USA.

^c^Molecular Neuropharmacology and Genetics Laboratory, Department of Neuroscience and Pharmacology, Faculty of Health and Medical Sciences, University of Copenhagen, Panum Institute 18.6, 2200 Copenhagen N, Denmark.

^d^Institute of Medical Chemistry, Medical University of Vienna, 1090 Vienna, Austria.

^e^Department of Pediatrics, The Saban Research Institute, Children's Hospital Los Angeles, Keck School of Medicine of USC, 4661 Sunset Blvd. Rm 307, Los Angeles, CA 90027, USA.

^h^Center for Addiction Research and Science - AddRess, Medical University Vienna, Waehringerstrasse 13A, 1090 Vienna, Austria.

*these authors equally contributed to the current work and are co-corresponding authors:

Prof. Dr. Harald H. Sitte:

[harald.sitte@meduniwien.ac.at](mailto:harald.sitte@meduniwien.ac.at), Tel: +43 1 40160 31323, FAX: +43 1 40160 931300; Center for Physiology and Pharmacology, Medical University of Vienna, Waehringerstraße 13A, 1090 Vienna, Austria

Prof. Dr. Lynette C. Daws

[daws@uthscsa.edu](mailto:daws@uthscsa.edu), Tel: 210 567 4361, FAX: 1 210 567 4410; University of Texas Health Science Center at San Antonio, Department of Cellular and Integrative Physiology, 7703 Floyd Curl Drive, MC7756, San Antonio, TX 78229-3900, United States of America

**Detailed Methods**

**1. EXPERIMENTAL MODEL DETAILS**

**OCT3+/+ and OCT3-/- mice**

Adult (>P60; 25–30 g) male wild-type mice (OCT3+/+), or OCT3 knock-out (KO, OCT3-/-) mice, bred on a C57BL/6 background, were obtained from an in house colony at The University of Texas Health Science Center at San Antonio (UTHSCSA). The mice were originally developed by Zwart and colleagues in 2001 (Zwart et al., 2001) and generously provided to us by Dr. Kim Tieu. These mice were used for all *in vivo* experiments (locomotor activity, chronoamperometry), and for quantitative autoradiography. Animals were housed in groups of five in a temperature-controlled (24°C) vivarium maintained on a 12/12-hr light/dark cycle (lights on at 7:00 am) in plastic cages (29cm x 18cm x 13 cm) containing rodent bedding (Sani-chips, Harlan Teklad, Madison, WI, USA) with free access to food (irradiated rodent sterilizable diet, Harlan Teklad) and water. Separate groups of mice were used for each different assay (locomotor activity, chronoamperometry, quantitative autoradiography). All procedures were conducted in accordance with the National Institute of Health Guide for the Care and Use of Laboratory Animals (Institute of Laboratory Animal Resources, Commission on Life Sciences, National Research Council 1996), and with the approval of the Institutional Animal Care and Use Committee, UTHSCSA.

Transgenic *Dat1*-eGFP mice which were generated by the GENSAT project (Gong et al., 2003). Cryopreserved embryos were obtained from Mutant Mouse Regional Resource Centre (strain name: Tg(Slc6a3-EGFP) JN119Gsat) and *Dat1*-eGFP mice were bred to C57BL/6 mice and used in the F1 generation. For the studies described here, only hemizygous *Dat1*-eGFP mice were used. Animals were housed under diurnal light conditions with food and water ad libitum, and mice of both genders were used in the experiments. All animal experiments were conducted in accordance with guidelines of the Danish Animal Experimentation Inspectorate (permission number: 2012-15-2934-00279). In total 24 mice were used (P4-P11).

**2. METHOD DETAILS**

**2.1. Isolation and mRNA expression analysis of dopaminergic neurons**

For isolation of DA neurons, we used transgenic *Dat1*-eGFP mice. Neurons were FAC sorted based on the expression of eGFP as previously described (Apuschkin et al., 2015). Isolated cells were collected on dry ice and kept at -80^o^C until further use. For each eGFP positive sample collected, an eGFP negative sorted sample of the approximately same cell number was collected simultaneously.

Isolated cells (8000-10000 cells) were lyzed and RNA extracted using Qiagen microRNA column according to the manufactures instructions, including DNAse treatment. RNA was converted to cDNA using SuperScript III (Thermofisher) (Reaction 1 (70^o^C 5 min): max 1µg RNA, 50ng random primer, 0.2 µmol DTT, H_2_O to a final volume 15 µl. Reaction 2 (10min 25^o^C, 50min 50^o^C, 5min 80^o^C); 10XRT buffer, 10nmol dNTP mix, 40U RNaseOUT, 200U Superscript, H_2_O to a final volume 5 µl, thereafter added to Reaction 1) and cDNA was diluted 10x with water before usage in qPCR reactions. qPCR was performed using an Agilent Mx3000p (Agilent Technologies) real-time thermocycler using SYBR green (PrecisionPlus 2x qPCR Mastermix, Primerdersign Ltd.) as probe (20 pmol primer forward, 20 pmol primer reverse, 7.4µl 2x PrecisionPlus qPCR mastermix, H_2_O to a final volume of 18 µl, 2 µl cDNA). Data was collected using MxPro software and analyzed using Microsoft excel and Graphpad prism software. Relative expression was calculated using the ΔΔC_q_ method as previously described (Pfaffl, 2001) using β-actin as reference gene for normalization. Samples were run in duplicates or triplicates and the average of these used for further analysis. For genes of interest, data was excluded for analysis if C_q_>35. For all primer solutions a 4*10x dilution series standard curve were performed in the same run as the genes of interest and the efficiency of the primer calculated using the following equation; Efficiency=-10^(-1/slope)^. No RT reactions for all genes of interest and primer solutions were performed and data excluded if the C_q_ value for the no RT reaction were within 5 cycles of the C_q_ value of the gene of interest. Intron spanning primers were designed using Primer3 and target specificity checked using PrimerBlast (http://www.ncbi.nlm.nih.gov/tools/primer-blast/). For visualization of detection of OCT3 in the samples, a fixed detection limit was set as Cq = 35, and the detection of OCT3 given as relative to this fixed detection limit using the following equation;

$Relative expression=\frac{{(1+E_{GOI})}^{{-\Delta C}_{q(GOI)}}}{2^{{-\Delta C}_{q(C_{q}=35)}}}$,

where

$\Delta C_{q}\left( C_{q}=35 \right)= 35-\left( \frac{C_{q}\left( \beta-\mathrm{actin}_{\mathrm{negative}} \right)+C_{q}\left( \beta-\mathrm{actin}_{\mathrm{positive}} \right)}{2} \right)$.

| Target/Gene of interest (GOI) | Forward | Reverse |
| --- | --- | --- |
| β-actin | TTCTACAATGAGCTGCGTGTG | GGGGTGTTGAAGGTCTCAAA |
| Dopamine transporter (*Dat*) | TGCTCTACTTCAGCCTGTGG | TATGCTCTGATGCCATCCAT |
| Tyrosine Hydroxylase (*Th*) | CCGTCATGCCTCCTCACCTATG | CCTGGGAGAACTGGGCAAATG |
| Vesicular monoamine transporter 2 (*Vmat2*) | GGTATGCTATCGGTCCCTCT | AGCCAAGGAAAGCCAAT |
| Nuclear receptor related 1 protein (*Nurr1*) | CACTACGGTGTTCGCACTTG | CATCCCAACAGCTAGGCACT |
| Glutamate decarboxylase (*Gad1*) | Atatcattggtttagctggtgaatg | Gtgactgtgttctgaggtgaagag |
| Organic cation transporter 3 (*Oct3*) | AGAAGGGATACCGTGGCTGA | AGCCTGAGCAGAGTGAAACC |

**2.2. Multiplex Fluorescence *In Situ* Hybridization (FISH)**

Fresh-frozen tissues from adult, male C57BL/6 mice were cryosectioned at 16 µm thickness, and stored at **-**80°C. Tissue was obtained from mice housed under the conditions described below. Commercially available RNAscope Multiplex Fluorescent reagent kits and RNAscope probes were used for transcript detection (Advanced Cell Diagnostics, Hayward, CA). RNAscope probe sets directly against *Vmat2*/*Slc18a2* (Cat. no. 425331), *Oct3*/*Slc22a3* (Cat. no. 439051-C2), and a negative control probe directly against a bacterial gene, *DapB* (Cat. No. 320871) were purchased from Advanced Cell Diagnostics, Hayward, CA. Each set of probes contained a tag that enables the target transcript to be visualized in a specific color channel (C): C1 for *Vmat2* probe set, excitation 495nm/emission 520 nm, C2 for *Oct3* probe set, excitation 555 nm/emission 575 nm, and C3 for *DapB* probe set, excitation 645nm/emission 670 nm. *In situ* hybridization was performed according to the manufacturer’s protocol. Briefly, slides were post-fixed with pre-chilled, freshly prepared 4% paraformaldehyde for 15 min at 4°C prior to dehydration with 50%, 70%, and 100% ethanol series at room temperature for five min each. Dehydrated slides were stored at **-**20°C until next day. After pretreating slides with Protease Pretreat 4 (Advanced Cell Diagnostics, Hayward, CA) at room temperature for 30 min, hybridization was performed at 40°C for two h in HybEZ oven (Advanced Cell Diagnostics, Hayward, CA). Following washing and amplification steps, coverslips were mounted on the slides with Prolong Gold antifade mounting medium with DAPI (Life Technology, CA).

Images were acquired as Z stacks using a Zeiss Axio Observer Inverted microscope fitted with a LSM700 confocal scanner (Cellular Imaging Core at the Saban Research Institute at Children’s Hospital Los Angeles) controlled by Zeiss Zen 2009 program. Figures were prepared digitally using Adobe Photoshop CS5.1 and Adobe Illustrator CS5.1 (Adobe Systems In., San Jose, CA).

**2.3. Locomotor activity**

**Locomotor activity**

Locomotor activity was measured using eight 30x15x15 cm acrylic boxes (Instrumentation Services, UTHSCSA) that were separately enclosed in sound-attenuating chambers (model no. ENV-022M, Med Associates, St. Albans, VT, USA). Four infrared light beams were spaced 6 cm apart and located 2 cm above the floor of each box. Occlusions of the beams were counted by Multi-Varimex version 1.00 software (Columbus Instruments, Columbus, OH, USA). The floor of the boxes consisted of a grid of parallel 2.3 mm stainless steel rods mounted 6.4 mm apart. Between tests, the floor and the inside of the boxes were wiped, and the litter paper beneath the floor was replaced. Each animal received an injection of saline or 0.1 mg/kg D22, and 60 min later, by an injection of saline or a particular dose of amphetamine (1, 3.2, or 10 mg/kg) (n=8 per treatment condition) after which locomotor activity was measured for 4 h. D22 and d-amphetamine sulfate, both obtained from Sigma-Aldrich Co. (St. Louis, Missouri, USA), were dissolved in physiological saline and injected intraperitoneally (i.p.) in a volume of 10 ml/kg. Doses are expressed as the weight of the salt.

Data were analyzed using GraphPad Prism version 6.05 (GraphPad.com) and NCSS 11 Statistical Software (2016). Locomotor activity was analyzed by multi-factor ANOVA followed by comparisons of means (Sidak’s test). P < 0.05 was considered statistically significant. Data are reported as mean ± standard error.

**2.4. High-speed Chronoamperometry**

High-speed chronoamperometry was conducted using the FAST-12 system (Quanteon; http://www.quanteon.cc) as previously described with minor modifications (Williams et al., 2007). Carbon fiber recording electrodes were constructed using a single carbon-fiber (30 μm diameter; Specialty Materials; http://www.specmaterials.com), which was sealed inside fused silica tubing (Schott, North America; <http://www.schott.com>). The exposed tip of the carbon fiber (150 μm in length) was coated with 5% Nafion (Aldrich Chemical Co.; htpp://www.sigmaaldrich.com; 3–4 coats baked at 200 °C for 5 min per coat) to provide a 1000-fold selectivity for DA over its metabolite dihydroxyphenylacetic acid (DOPAC). Under these conditions, microelectrodes displayed linear amperometric responses to 0.5–10 μM DA during *in vitro* calibration in 100 mM phosphate-buffered saline (pH 7.4).

To locally deliver drugs close to the recording site, a multi-barrelled micropipette (FHC; http://www.fh-co.com) was positioned adjacent to the microelectrode using sticky wax (Moyco; http://www.moycotech.com). The distance between the microelectrode and micropipette tips was 200 μm. Barrels of the micropipette were filled with either amphetamine (400 µM; Sigma), cocaine (400 µM; Sigma), D22 (10 µM; Sigma) or vehicle (aCSF). Mice were anesthetized by i.p. injection (2 ml/kg body weight) of a mixture of urethane (250 mg/kg) and α-chloralose (25 mg/kg), followed by tracheal intubation to facilitate breathing, and placed into a stereotaxic frame (David Kopf Instruments; http://www.kopfinstruments.com). The electrode/micropipette assembly was lowered into the striatum at the following coordinates (in mm) from bregma: A/P + 1.1; M/L +/- 1.4; D/V -2.25mm to -2.70. Body temperature was maintained at 36-37 °C and blood oxygen levels monitored (MouseOximeter, StarrLifeSciences) and maintained above 90%.

Drugs were pressure-ejected into striatum using a Picospritzer II (General Valve Corporation; http://www.parker.com) in an ejection volume of 125 nl (5–25 psi for 0.25–3 s) according to the following sequence: First, amphetamine (50 pmol) was pressure-ejected, producing robust DA release ranging from ~0.5 to 2.0 micromolar peak signal amplitude. A period of 45 min was permitted to elapse to allow releasable pools of DA to be restored (Owens et al., 2012). Then either D22 (1.25 pmol), cocaine (50 pmol), a combination of D22 (1.25 pmol) and cocaine (50 pmol), or vehicle was pressure-ejected. In all instances two intrastriatal ejections were made so as to control for the D22+cocaine combination. Ejection combinations, each separated by two min, are as follows: aCSF + aCSF, D22 + aCSF, aCSF + cocaine and D22 + cocaine. Note that none of these solutions produced an electrochemical signal themselves, nor did they interfere with the recording properties of the electrode at the concentrations used here (see (Baganz et al., 2008, Davidson et al., 2000)). Fifteen min later, the same pmol amount of amphetamine was again pressure-ejected, and then again at 60 and 105 min following drug administration.

Note that after ejection of drugs, there is an estimated 10-200-fold dilution caused by diffusion through the extracellular matrix to reach a concentration of 2-40 μM (for amphetamine and cocaine) or 0.05-5 μM (for D22) at the recording electrode (Callaghan et al., 2005). The concentration range for amphetamine and cocaine is consistent with those measured in brain following behaviorally relevant doses of these drugs (Clausing et al., 1995, Zombeck et al., 2009). We found that behaviorally effective doses of D22 (0.1 mg/kg) gives rise to brain concentrations in the nanomolar range ((Horton et al., 2013) and unpublished data). To record the efflux and clearance of DA at the active electrode, oxidation potentials consisting of 100 ms pulses of 550 mV, each separated by a 900 ms interval during which the resting potential was maintained at 0 mV, were applied with respect to a Ag/AgCl reference electrode implanted into the contralateral superficial cortex. Oxidation and reduction currents were digitally averaged during the last 80 ms of each 100 ms voltage pulse. For each recording session, DA was identified by its reduction/oxidation current ratio: 0.50–0.90.

At the conclusion of each experiment, an electrolytic lesion was made to mark the placement of the recording electrode tip. Mice were then decapitated while still anesthetized, and their brains removed, frozen on dry ice, and stored at −80°C until sectioned (20 μm) for histological verification of electrode location within the striatum. Data were analyzed using GraphPad Prism version 6.05. In addition to reduction/oxidation ratios, five signal parameters were analyzed: (i) maximal signal amplitude of amphetamine-evoked DA release (in μM); (ii) time to reach maximal signal amplitude (rise time, in s); (iii) DA efflux rate (in nM/s), which is the change in DA oxidation current evoked by amphetamine application as a function of time; (iv) clearance time, the time for released DA to be cleared by 80% of maximal amplitude (T_80_, in s); and (v) DA clearance rate (in nM/s), defined as the slope of the linear portion of the current decay curve, i.e., from 20−60% of maximal signal amplitude. Differences in baseline signal parameters for amphetamine-evoked DA release between genotypes were analyzed by t-tests for independent samples (Supplementary Table S1). Percent change in amphetamine-evoked release following vehicle/drug combinations were analyzed by two-way ANOVA followed by Fisher’s LSD (Figure 3, main article) or two-tailed Mann Whitney tests (Supplementary Table S2). Within genotype, data for pre- versus post-vehicle/drug effects on amphetamine-evoked DA release were analyzed by paired t-tests (Supplementary Table S3). All data are presented as mean and standard error of the mean (S.E.M.), unless indicated otherwise. P < 0.05 was considered statistically significant.

**2.5. [^3^H]WIN35428 binding to dopamine transporters**

OCT3+/+ and OCT3-/- mice not previously used for other experiments were killed by decapitation and brains rapidly removed and frozen on powdered dry ice before being stored at -80 °C until sectioned for quantitative autoradiography. Brains were brought to -20 °C in a cryostat (Leica CM 1850, Meyer Instruments, Houston, TX) and coronal sections (20 µm) thaw mounted onto gelatin coated microscope slides, desiccated for 18-24 h at 4°C and frozen at -80°C until use. Dopamine transporter (DAT) binding was performed following the methods of described in (Galici et al., 2003). Sections were defrosted for 10 min at 4°C, pre-washed in 30 mM sodium phosphate buffer, pH 7.4 for 1 h. Incubation with 12 nM [^3^H]WIN35428 at 4°C in pH 7.4 buffer was carried out for 2 h. GBR12909 (25 µM) was used to define non-specific binding in adjacent sections on separate slides. Sections were washed twice for 1 min each in 4°C pH 7.4 buffer, dipped for two s in 4°C deionized water, and dried on a slide warmer at a moderate heat setting for 20 min. Sections on slides were opposed to Kodak Biomax MR film for 6 weeks along with [^3^H] standards calibrated to labeled brain mash to obtain units of fmol/mg pr (Geary et al., 1985). Autoradiographic images were captured on a digital imaging station (Northern Lights illuminator, Scion Image CCD camera, copy stand), calibrated using a linear function and binding density was measured using Image J software (<http://rsb.info.nih.gov/ij/download.html>). Data were analyzed using GraphPad Prism version 6.05. Differences in specific [^3^H]WIN35428 between genotypes were analyzed by t-tests for independent samples (Supplementary Table S 4).

**2.6. Radiotracer-flux studies in superior cervical ganglion (SCG) cells:**

Superior cervical ganglion (SCG) culture preparation was performed as described earlier (Salzer et al., 2014, Kristufek et al., 2002). Rats were sacrificed by decapitation after short CO_2_ asphyxia in accordance with the ARRIVE guidelines and the Austrian animal protection law (see [http://www.ris.bka.gv.at/Dokumente/BgblAuth/BGBLA_2012_I_114/BGBLA_2012_I_114.pdf](https://webmail.meduniwien.ac.at/#NOP)) and the Austrian animal experiment by-laws (see [http://www.ris.bka.gv.at/Dokumente/BgblAuth/BGBLA_2012_II_522/BGBLA_2012_II_522.pdf](https://webmail.meduniwien.ac.at/#NOP)) which implement European (DIRECTIVE 2010/63/EU; see [http://eur-lex.europa.eu/LexUriServ/LexUriServ.do?uri=OJ:L:2010:276:0033:0079:en:PDF](https://webmail.meduniwien.ac.at/#NOP)) in Austrian law. Collected ganglia were incubated in collagenase (1.5 mg ml^-1^, Sigma-Aldrich) and dispase (3.0 mg ml^-1^, Boehringer Mannheim, Vienna, Austria) for 30 min followed by 0.25% trypsin (Worthington, Lakewood, NJ, USA) for 15 min at 37ºC. After trituration, cells were resuspended in DMEM containing 4.5 g l^-1^ glucose, 10 mg l^-1^ insulin, 25 000 IU l^-1^ penicillin, 25 mg l^-1^ streptomycin (all Sigma-Aldrich), 50 µg l^-1^ nerve growth factor (Biomedica, Vienna, Austria), and 5% heat-inactivated fetal calf serum (Biochrom, Berlin, Germany) and seeded onto 35 mm culture dishes coated with poly D-lysine (Sigma-Aldrich). Cultures were kept at 37ºC in a humidified 5% CO_2_ atmosphere. Radiotracer-flux studies in SCG cells were analyzed using Microsoft excel and GraphPad Prism.

**2.7. Radiotracer-flux studies in HEK293 cells**

Generation of human embryonic kidney 293 (HEK293) cells stably expressing human norepinephrine transporter (hNET) or yellow-fluorescent protein-tagged human organic cation transporter 3 (YFPhOCT3) was performed as described earlier (Scholze et al., 2002). Cells were maintained in humidified atmosphere at 37°C, 5% CO_2_ in Dulbecco’s modified Eagle’s medium (DMEM), supplemented with 10% fetal calf serum (Invitrogen).

*i) Uptake inhibition*: Cells expressing the desired transporter were seeded at a density of 40,000 cells/well in poly-d-lysine (PDL) coated 96-well plates one day prior to the experiment. As described elsewhere (Mayer et al., 2017, Scholze et al., 2000), the specific activity of the radiolabeled substrate (1-methyl-4-phenylpyridinium, MPP^+^) was kept constant (20 nM [^3^H]MPP^+^). Briefly, the cells were washed with Krebs- HEPES-Buffer (KHB, 120 mM NaCl, 3 mM KCl, 2 mM CaCl_2_, 2 mM KgCl_2_, 2 mM d-glucose monohydrate, 10 mM HEPES, pH 7.3) and pre-incubated for five min with increasing concentrations of the substance of interest. After five min, [^3^H]MPP^+^ was added and the uptake was terminated after three min. Subsequently, cells were lysed in 1% SDS and radioactivity was quantified by liquid scintillation counting. Data were normalized to the absolute counts per min in absence of inhibitors (100 %). Non-specific uptake was determined in presence of 100 µM decynium-22 (D22) (YFPhOCT3) or 10 µM mazindole (hNET).

*ii) Release studies:* HEK293 cells expressing hNET or YFPhOCT3 and human vesicular monoamine transporter 2 (hVMAT2) were grown on PDL-coated glass coverslips (5mm diameter, 40.000 cells/coverslip) overnight. According to earlier studies (Scholze et al., 2002), cells were pre-loaded with [^3^H]MPP^+^ (0.1 µM, 30 min at 37°C), transferred into small chambers (volume: 200 µl) and superfused with KHB. After establishment of a stable baseline, two-min fractions of superfusates were collected and the cells were treated with various substances or vehicle after three basal fractions. Finally, cells were lysed with 1% SDS and radioactivity was determined by liquid scintillation counting. The release of [^3^H]MPP^+^ was expressed as fractional rate, *i.e.* the amount of radioactivity released per two-min fraction was expressed as percentage of total radioactivity present at the beginning of that fraction (Sitte et al., 2000). Amphetamine induced release was calculated by subtracting the mean of the first three basal fractions from the mean of the last four fractions in presence of amphetamine.

Radiotracer-flux studies in YFPhOCT3 cells were analyzed using Microsoft excel and GraphPad Prism (GraphPad.com).

**2.8. Uptake and release of ASP^+^ in YFPhOCT3 cells**

YFPhOCT3 expressing cells were grown on CELLVIEW™ cell culture dishes (Greiner Bio-one, Kremsmuenster, Austria) and superfused with KHB containing the substances of interest, directly applied onto the cells by use of a microsuperfusion pipette (Octaflow™, ALA Scientific Instruments Inc., Farmingdale, NY, USA). To avoid the accumulation of non-specific fluorescence a constant flow of KHB (2.5 ml /min, 25°C) was maintained by a macro‐superfusion system. ASP^+^ was excited with a pulsed high‐power bluewhite LED (CREE Inc., Racine WI USA), set to 480 nm and the emission was continuously recorded at 609 nm using a high sensitivity photomultiplier tube photo‐detector (HAMAMATSU Photonics, Hersching am Ammersee, Germany) system of a reconfigured Sequoia‐Turner 450 fluorometer adapted to an inverted epiflourescence microscope (Olympus IX50, Olympus, Tokyo, Japan). YFPhOCT3 expressing cells were superfused with KHB for 20 s to establish the basal background fluorescence. Afterwards, 3 µM ASP^+^ was added at (t=20 s) for 40 s to record the initial untreated uptake rate. Subsequently, the superfusion system was switched to 3 µM ASP^+^ (at t=60 s) supplemented with the substance of interest for 140 s. For analysis, the background-fluorescence (RFU) at t=20 s was subtracted from each individual trace. In addition, each trace was normalized to the relative fluorescent units at t=60. Uptake and release of ASP^+^ in YFPhOCT3 cells was analyzed using pClamp10.3, Microsoft excel and GraphPad Prism.

**2.9. Drugs and Chemicals**

*S*(+)-Amphetamine sulfate (termed amphetamine throughout the paper) and decynium-22 (D22), both obtained from Sigma-Aldrich Co. (St. Louis, Missouri, USA), were dissolved in physiological saline and injected i.p. in a volume of 10 ml/kg. Doses were expressed as the weight of the salt. ASP^+^, reserpine, 1-methyl-4-phenylpyridinium (MPP^+^), cocaine, corticosterone were also obtained from Sigma-Aldrich Co (St. Louis, Missouri, USA). [^3^H]WIN35,428 was obtained from Perkin Elmer (Waltham, MA, USA; specific activity: 76 Ci/mmol). [^3^H]MPP^+^ was obtained from ARC (St. Louis, MO, USA; specific activity: 80 Ci/mmol).

**3. QUANTIFICATION AND STATISTICAL ANALYSIS**

Calculations were performed using Microsoft Excel® 2010 (Microsoft Corporation, Redmond, Washington, USA), GraphPad Prism 5.0. or above (GraphPad Software Inc., La Jolla, California, USA), and NCSS 11 Statistical Software (2016), as indicated. Statistical tests were used wherever appropriate. Statistical details of experiments can be found in the figure legends.

**4. Supplementary Figures**

**Supplementary Figure S1.**

**
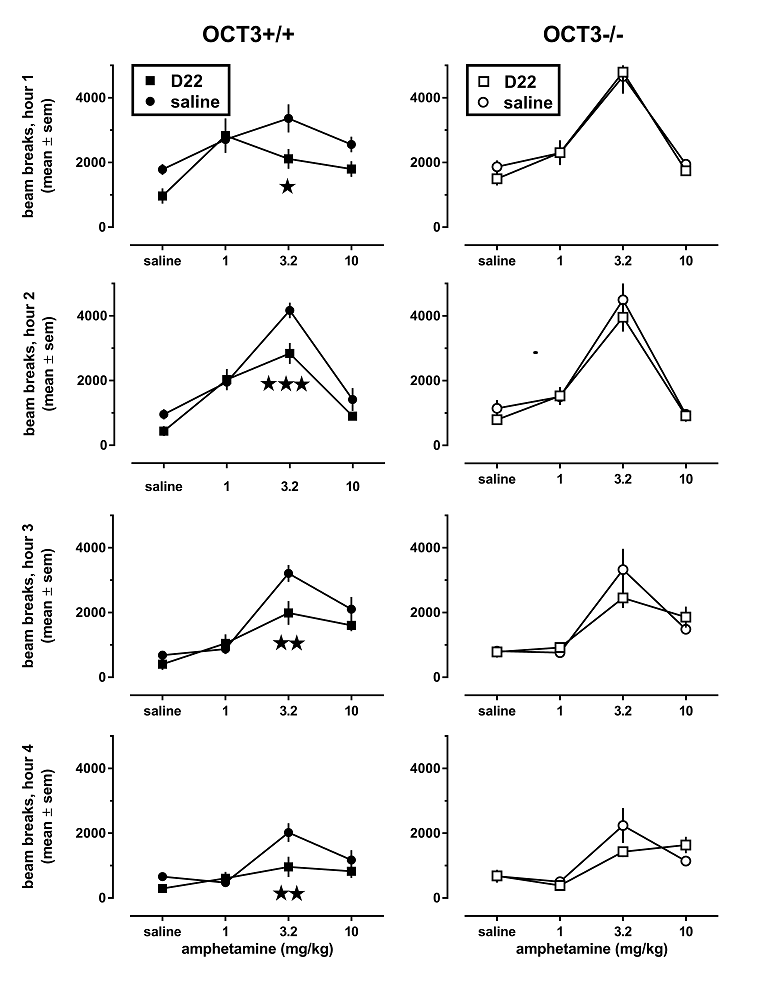
**

**Supplementary Figure S1: Dose- and time-dependent effects of D22 on amphetamine-induced locomotion in OCT3+/+ and OCT3-/- mice.**

In animals pretreated with saline (filled and open circles), amphetamine increased locomotion in a dose- and time-dependent manner, and did so similarly in both genotypes. A three-factor ANOVA with Geisser-Greenhouse adjustments showed significant main effects of amphetamine dose [F(3,56)=37.6, p<0.0001] and time [F(3,168)=109, p<0.0001], a significant interaction between amphetamine dose and time [F(3,168)=14.0, p<0.0001], but no statistically significant main or interaction effects involving genotype. In contrast, in animals pretreated with D22 (filled and open squares), the effects of amphetamine differed between genotypes, as evidenced by significant interactions between amphetamine and genotype [F(3,55)=3.79, p=0.015] and between amphetamine, genotype and time [F(9,165)=4.94, P=0.00015]. Results obtained in animals pretreated with D22 or saline were compared for each genotype and time period by two-factor ANOVA followed by Sidak-adjusted multiple comparisons. Statistically significant differences between locomotion in D22- and saline-pretreated animals were observed at 3.2 mg/kg amphetamine at all time intervals in OCT3+/+ animals, but not in OCT3-/- animals: * p < 0.05, ** p<0.01, *** p < 0.005. Results are shown as mean ± S.E.M. (n=7-8; error bars that are not shown are contained within the symbol).

**Supplementary Figure S2.**

**Supplementary Figure S2: Inhibition of [^3^H]MPP^+^ uptake via YFPhOCT3 by corticosterone and D22 but not cocaine and amphetamine**

Cells expressing YFPhOCT3 were incubated with increasing concentrations (1 nM to 1 mM) of **(A)**cocaine (COC) or amphetamine (AMPH) or (**B)** corticosterone (CORT) or D22. After 5 min, 20 nM [^3^H]MPP^+^ was added and uptake was terminated after three min by washing the cells with ice-cold buffer. Non-specific uptake was determined in the presence of 100 µM D22 and subtracted. Uptake is represented as percent of uptake in absence of inhibitor. Symbols are mean ± S.E.M. obtained from three experiments performed in triplicate.

**Supplementary Table S1.** **Comparison of amphetamine-evoked DA release signal parameters in striatum of OCT3+/+ and OCT3-/- mice**

|  | Peak DA Release (μM) | Rise Time  (s) | Release Rate  (nM/s) | Clearance Time, T_80_ (s) | Clearance Rate  (nM/s) |
| --- | --- | --- | --- | --- | --- |
| OCT3+/+ (n=37) | 1.00 ± 0.16 | 227 ± 45 | 9.2 ± 1.4 | 646 ± 83 | 4.2 ± 1.7 |
| OCT3-/- (n=36) | 0.80 ± 0.11 | 221 ± 35 | 7.0 ± 1.3 | 771 ± 120 | 2.1 ± 0.6 |

There were no significant differences in signal parameters for baseline amphetamine-evoked DA release between OCT3+/+ and OCT3-/- mice. Amphetamine-evoked release in striatum was predominantly DA, as evident by reduction/oxidation current ratios being greater than 0.50 and were 0.54 ± 0.03 and 0.66 ± 0.04 for OCT3+/+ and OCT3-/- mice respectively. Data are mean and S.E.M.

**Supplementary Table S2.** **Effect of vehicle, D22 and cocaine on amphetamine-evoked DA release and clearance parameters**

|  | **aCSF + aCSF** | **D22 + aCSF** | **aCSF + Cocaine** | **D22 + Cocaine** |
| --- | --- | --- | --- | --- |
| **Peak DA Release** | | | | |
| OCT3+/+  OCT3-/- | 118 ± 15 (8)  89 ± 15 (8) | 59 ± 7 (8)*  88 ± 16 (14) | 77 ± 15 (11)*  42 ± 9 (7)* | 42 ± 9 (10)*  40 ± 7 (7)* |
| **Rise Time** | | | | |
| OCT3+/+  OCT3-/- | 111 ± 23  105 ± 33 | 156 ± 46  112 ± 20 | 162 ± 57  79 ± 10 | 228 ± 160  301 ± 155 |
| **Rise Rate** | | | | |
| OCT3+/+  OCT3-/- | 128 ± 24  167 ± 70 | 77 ± 28  146 ± 65 | 109 ± 39  64 ± 17 | 149 ± 72  51 ± 19 |
| **Clearance Time (T_80_)** | | | | |
| OCT3+/+  OCT3-/- | 194 ± 72  185 ± 63 | 149 ± 34  157 ± 31 | 140 ± 60  31 ± 12* | 97 ± 40  208 ± 97 |
| **Clearance Rate** | | | | |
| OCT3+/+  OCT3-/- | 119 ± 60  104 ± 35 | 85 ± 55  108 ± 56 | 186 ± 74  228 ± 69 | 204 ±110  37 ± 14 |

As expected, aCSF vehicle had no significant effect on any of the signal parameters in either genotype. Under the conditions and doses used here, the signal parameter most sensitive to drug effects was peak DA release (see Figure 3 main article and Supplementary Table 3). Consistent with the well-known role of DAT in amphetamine’s actions, the DAT blocker cocaine blunted amphetamine-evoked DA release in both OCT3+/+ (by ~23%) and OCT3-/- mice (by ~58%). Though the effect size tended to be larger in OCT3-/- mice, there was no significant difference between genotypes. Data are mean and S.E.M. *percent* change from baseline amphetamine-evoked DA signal (see Supplementary Table 3 for absolute data values). Numbers in parentheses represent sample size. * P < 0.05, two-tailed Mann Whitney compared to genotype-matched control group.

**Supplementary Table S3.** **Comparison of amphetamine-evoked DA release signal parameters in striatum of OCT3+/+ and OCT3-/- mice, before and after combinations of vehicle, D22 and cocaine.**

|  | **aCSF + aCSF** | | **D22 + aCSF** | | **aCSF + Cocaine** | | **D22 + Cocaine** | |
| --- | --- | --- | --- | --- | --- | --- | --- | --- |
|  | Pre- | Post- | Pre- | Post- | Pre- | Post- | Pre- | Post- |
| **Peak DA Release (µM)** | | | | | | | | |
| OCT3+/+  OCT3-/- | 0.55 ± 0.14  0.61 ± 0.09 | 0.63 ± 0.14  0.50 ± 0.08 | 2.00 ± 0.51  1.16 ± 0.22 | 1.01 ± 0.21*  0.82 ± 0.15 | 0.69 ± 0.08  0.44 ± 0.10 | 0.49 ± 0.10*  0.19 ± 0.07* | 0.91 ± 0.24  0.64 ± 0.20 | 0.35 ± 0.15*  0.27 ± 0.11* |
| **Rise Time (s)** | | | | | | | | |
| OCT3+/+  OCT3-/- | 204 ± 81  315 ± 99 | 387 ± 206  268 ± 98 | 153 ± 22  226 ± 61 | 221 ± 87  202 ± 47 | 247 ± 109  217 ± 55 | 246 ± 93  163 ± 51 | 283 ± 100  108 ± 22 | 142 ± 35  234 ± 85 |
| **Rise Rate (nM/s)** | | | | | | | | |
| OCT3+/+  OCT3-/- | 4.16 ± 0.87  5.84 ± 2.35 | 4.81 ± 1.14  6.67 ± 3.01 | 13.21 ± 2.46  9.04 ± 2.49 | 10.46 ± 4.17  6.67 ± 1.79 | 9.08 ± 2.40  4.59 ± 2.59 | 7.14 ± 3.11  2.65 ± 1.74 | 10.13 ± 3.82  6.58 ± 1.80 | 2.49 ± 0.55  3.20 ± 1.76 |
| **Clearance Time, T_80_ (s)** | | | | | | | | |
| OCT3+/+  OCT3-/- | 544 ± 139  536 ± 148 | 599 ± 135  783 ± 270 | 660 ± 162  924 ± 258 | 860 ± 211  891 ± 181 | 629 ± 146  829 ± 235 | 576 ± 142  210 ± 51* | 734 ± 216  678 ± 206 | 443 ± 137  964 ± 417 |
| **Clearance Rate (nM/s)** | | | | | | | | |
| OCT3+/+  OCT3-/- | 0.99 ± 0.27  4.34 ± 2.28 | 1.70 ± 0.82  1.49 ± 0.55 | 10.45 ± 7.15  1.94 ± 0.37 | 4.55 ± 3.52  1.14 ± 0.30 | 2.93 ± 1.15  0.81 ± 0.31 | 3.18 ± 1.61  1.03 ± 0.19 | 3.26 ± 1.96  1.17 ± 0.22 | 1.02 ± 0.42  0.33 ± 0.13* |
| **Red:Ox** | | | | | | | | |
| OCT3+/+  OCT3-/- | 0.54 ± 0.09  0.65 ± 0.06 | 0.58 ± 0.09  0.58 ± 0.04 | 0.63 ± 0.03  0.77 ± 0.05 | 0.62 ± 0.04  0.74 ± 0.05 | 0.52 ± 0.05  0.53 ± 0.11 | 0.50 ± 0.06  0.35 ± 0.12* | 0.54 ± 0.07  0.59 ± 0.07 | 0.43 ± 0.11  0.44 ± 0.12 |

Data are mean and S.E.M. * P < 0.05 two-tailed paired t-test comparing pre- vs post- signal parameter within genotype. For wild-type mice, n = 8, 8, 11 and 10; and for OCT3-/- mice, n = 8, 14, 7 and 7 for aCSF + aCSF, D22 + aCSF, aCSF + Cocaine and D22 + Cocaine, respectively.

Recapitulating reports of others and our own findings (Zahniser et al., 1999, Daws et al., 2002), in OCT3+/+ mice DA clearance rate after cocaine given by itself tended to increase, consistent with cocaine inducing mobilization of DAT to the plasma membrane. Thus, when plasma membrane DATs are not fully blocked by cocaine and extracellular concentrations of DA are not sufficiently high to cause DAT to operate at maximal capacity (i.e. maximal velocity for DA clearance has not yet been reached), a “paradoxical” increase in DA clearance rate results (Zahniser et al., 1999, Daws et al., 2002). In addition, these data also support the idea that non-DAT transporters, such as OCTs and the plasma membrane monoamine transporter (PMAT), which have a high-capacity to transport DA and other biogenic amines (Engel et al., 2004, Dahlin et al., 2007, Daws, 2009), are able to effectively clear DA when DAT function is pharmacologically compromised.

Lack of significant effects of cocaine and D22 on rise rate seems counterintuitive at first glance. However, given that the concentrations of cocaine and D22 used here were selected so as not to produce maximal effects when given alone (i.e. to permit detection of additive effects of D22 and cocaine when given together) this is perhaps not surprising. While the amount of DA release would be expected to be less (fewer transporters in inward-facing conformation for DA to escape through), the rate of release would not necessarily be slower. Certainly in some cases there was a clear attenuation of DA release rate (e.g. see representative signal traces for the effect of D22 on amphetamine-evoked DA release in OCT3+/+ mice and the effect of cocaine on amphetamine-evoked DA release in OCT3-/- mice in Figure 3A, main article), however this occurred less often than not.

It is worth noting the relatively high degree of variance associated with most signal parameters in our chronoamperometric measurements: This is neither surprising nor unusual. A primary factor contributing to this variance is the excellent spatial resolution afforded by these small carbon fiber recording electrodes. Thus, the extracellular milieu surrounding the electrode (e.g. density of transporters) may differ significantly from experiment to experiment (*i.e.*, from mouse to mouse), regardless of how consistent stereotaxic placement may be. Similarly, factors affecting diffusion through extracellular fluid (such as tortuosity and volume fraction) may also contribute to variance in release and clearance kinetics from one experiment to the next (compare representative pre-drug DA signals in Figure 3A). Thus, our ability to detect marked effects of D22 and cocaine on amphetamine-evoked DA release in the face of this inherent “noise” underscores the magnitude and robustness of these drug effects on amphetamine-evoked DA release.

**Supplementary Table S4.** **DAT expression does not differ between OCT3+/+ and OCT3-/- mice**

|  | Dorsal Striatum | Ventral Striatum | Nucleus Accumbens Core | Nucleus Accumbens Shell |
| --- | --- | --- | --- | --- |
| OCT3+/+ (n=6) | 339 ± 38 | 444 ± 21 | 358 ± 15 | 201 ± 29 |
| OCT3-/- (n=5) | 338 ± 19 | 464 ± 20 | 362 ± 12 | 173 ± 21 |

To investigate the possibility that DAT expression may be greater in OCT3-/- mice, to compensate for constitutive loss of OCT3, we used quantitative autoradiography to measure DAT density in brain using the DAT selective ligand [^3^H]WIN35428. Specific [^3^H]WIN35428 binding in terminal regions (striatum and nucleus accumbens) and cell body regions (ventral tegmental area, substantia nigra) did not differ between genotypes. Data are mean ± S.E.M. fmol/mg protein.

**Supplementary References**

APUSCHKIN, M., STILLING, S., RAHBEK-CLEMMENSEN, T., SORENSEN, G., FORTIN, G., HERBORG HANSEN, F., ERIKSEN, J., TRUDEAU, L. E., EGEROD, K., GETHER, U. & RICKHAG, M. 2015. A novel dopamine transporter transgenic mouse line for identification and purification of midbrain dopaminergic neurons reveals midbrain heterogeneity. *Eur J Neurosci,* 42**,** 2438-54.

BAGANZ, N. L., HORTON, R. E., CALDERON, A. S., OWENS, W. A., MUNN, J. L., WATTS, L. T., KOLDZIC-ZIVANOVIC, N., JESKE, N. A., KOEK, W., TONEY, G. M. & DAWS, L. C. 2008. Organic cation transporter 3: Keeping the brake on extracellular serotonin in serotonin-transporter-deficient mice. *Proc Natl Acad Sci U S A,* 105**,** 18976-81.

CALLAGHAN, P. D., IRVINE, R. J. & DAWS, L. C. 2005. Differences in the in vivo dynamics of neurotransmitter release and serotonin uptake after acute para-methoxyamphetamine and 3,4-methylenedioxymethamphetamine revealed by chronoamperometry. *Neurochem Int,* 47**,** 350-61.

CLAUSING, P., GOUGH, B., HOLSON, R. R., SLIKKER, W., JR. & BOWYER, J. F. 1995. Amphetamine levels in brain microdialysate, caudate/putamen, substantia nigra and plasma after dosage that produces either behavioral or neurotoxic effects. *J Pharmacol Exp Ther,* 274**,** 614-21.

DAHLIN, A., XIA, L., KONG, W., HEVNER, R. & WANG, J. 2007. Expression and immunolocalization of the plasma membrane monoamine transporter in the brain. *Neuroscience,* 146**,** 1193-211.

DAVIDSON, C., ELLINWOOD, E. H., DOUGLAS, S. B. & LEE, T. H. 2000. Effect of cocaine, nomifensine, GBR 12909 and WIN 35428 on carbon fiber microelectrode sensitivity for voltammetric recording of dopamine. *J Neurosci Methods,* 101**,** 75-83.

DAWS, L. C. 2009. Unfaithful neurotransmitter transporters: focus on serotonin uptake and implications for antidepressant efficacy. *Pharmacol Ther,* 121**,** 89-99.

DAWS, L. C., CALLAGHAN, P. D., MORON, J. A., KAHLIG, K. M., SHIPPENBERG, T. S., JAVITCH, J. A. & GALLI, A. 2002. Cocaine increases dopamine uptake and cell surface expression of dopamine transporters. *Biochem Biophys Res Commun,* 290**,** 1545-50.

ENGEL, K., ZHOU, M. & WANG, J. 2004. Identification and characterization of a novel monoamine transporter in the human brain. *J Biol Chem,* 279**,** 50042-9.

GALICI, R., GALLI, A., JONES, D. J., SANCHEZ, T. A., SAUNDERS, C., FRAZER, A., GOULD, G. G., LIN, R. Z. & FRANCE, C. P. 2003. Selective decreases in amphetamine self-administration and regulation of dopamine transporter function in diabetic rats. *Neuroendocrinology,* 77**,** 132-40.

GEARY, W. A., 2ND, TOGA, A. W. & WOOTEN, G. F. 1985. Quantitative film autoradiography for tritium: methodological considerations. *Brain Res,* 337**,** 99-108.

GONG, S., ZHENG, C., DOUGHTY, M. L., LOSOS, K., DIDKOVSKY, N., SCHAMBRA, U. B., NOWAK, N. J., JOYNER, A., LEBLANC, G., HATTEN, M. E. & HEINTZ, N. 2003. A gene expression atlas of the central nervous system based on bacterial artificial chromosomes. *Nature,* 425**,** 917-25.

HORTON, R. E., APPLE, D. M., OWENS, W. A., BAGANZ, N. L., CANO, S., MITCHELL, N. C., VITELA, M., GOULD, G. G., KOEK, W. & DAWS, L. C. 2013. Decynium-22 enhances SSRI-induced antidepressant-like effects in mice: uncovering novel targets to treat depression. *J Neurosci,* 33**,** 10534-43.

KRISTUFEK, D., RUDORFER, W., PIFL, C. & HUCK, S. 2002. Organic cation transporter mRNA and function in the rat superior cervical ganglion. *J Physiol,* 543**,** 117-34.

MAYER, F. P., LUF, A., NAGY, C., HOLY, M., SCHMID, R., FREISSMUTH, M. & SITTE, H. H. 2017. Application of a Combined Approach to Identify New Psychoactive Street Drugs and Decipher Their Mechanisms at Monoamine Transporters. *Curr Top Behav Neurosci,* 32**,** 333-350.

OWENS, W. A., WILLIAMS, J. M., SAUNDERS, C., AVISON, M. J., GALLI, A. & DAWS, L. C. 2012. Rescue of dopamine transporter function in hypoinsulinemic rats by a D2 receptor-ERK-dependent mechanism. *J Neurosci,* 32**,** 2637-47.

PFAFFL, M. W. 2001. A new mathematical model for relative quantification in real-time RT-PCR. *Nucleic Acids Res,* 29**,** e45.

SALZER, I., GAFAR, H., GINDL, V., MAHLKNECHT, P., DROBNY, H. & BOEHM, S. 2014. Excitation of rat sympathetic neurons via M1 muscarinic receptors independently of Kv7 channels. *Pflugers Arch,* 466**,** 2289-303.

SCHOLZE, P., NORREGAARD, L., SINGER, E. A., FREISSMUTH, M., GETHER, U. & SITTE, H. H. 2002. The role of zinc ions in reverse transport mediated by monoamine transporters. *J Biol Chem,* 277**,** 21505-13.

SCHOLZE, P., ZWACH, J., KATTINGER, A., PIFL, C., SINGER, E. A. & SITTE, H. H. 2000. Transporter-mediated release: a superfusion study on human embryonic kidney cells stably expressing the human serotonin transporter. *J Pharmacol Exp Ther,* 293**,** 870-8.

SITTE, H. H., SCHOLZE, P., SCHLOSS, P., PIFL, C. & SINGER, E. A. 2000. Characterization of carrier-mediated efflux in human embryonic kidney 293 cells stably expressing the rat serotonin transporter: a superfusion study. *J Neurochem,* 74**,** 1317-24.

WILLIAMS, J. M., OWENS, W. A., TURNER, G. H., SAUNDERS, C., DIPACE, C., BLAKELY, R. D., FRANCE, C. P., GORE, J. C., DAWS, L. C., AVISON, M. J. & GALLI, A. 2007. Hypoinsulinemia regulates amphetamine-induced reverse transport of dopamine. *PLoS Biol,* 5**,** e274.

ZAHNISER, N. R., LARSON, G. A. & GERHARDT, G. A. 1999. In vivo dopamine clearance rate in rat striatum: regulation by extracellular dopamine concentration and dopamine transporter inhibitors. *J Pharmacol Exp Ther,* 289**,** 266-77.

ZOMBECK, J. A., GUPTA, T. & RHODES, J. S. 2009. Evaluation of a pharmacokinetic hypothesis for reduced locomotor stimulation from methamphetamine and cocaine in adolescent versus adult male C57BL/6J mice. *Psychopharmacology (Berl),* 201**,** 589-99.

ZWART, R., VERHAAGH, S., BUITELAAR, M., POPP-SNIJDERS, C. & BARLOW, D. P. 2001. Impaired activity of the extraneuronal monoamine transporter system known as uptake-2 in Orct3/Slc22a3-deficient mice. *Mol Cell Biol,* 21**,** 4188-96.
